# CHEESE and WHEY: The Outcome of Milk Curdling

**DOI:** 10.3390/foods10051008

**Published:** 2021-05-05

**Authors:** Golfo Moatsou, Ekaterini Moschopoulou

**Affiliations:** Laboratory of Dairy Research, Department of Food Science and Human Nutrition, Iera Odos 75, 11855 Athens, Greece; catmos@aua.gr

The present Special Issue is dedicated to both products of the cheesemaking process, that is cheese and whey. Cheese is an excellent and complex food matrix that preserves in concentrated form valuable milk constituents, such as proteins, minerals, vitamins, and biofunctional lipids. The formation of cheese mass requires the removal of whey, i.e., water and soluble milk substances—proteins, minerals, lactose, and vitamins—. According to Fox and McSweeney [1], cheese is the most diverse group of dairy products manufactured from a few kinds of milk by means of a protocol that is more or less common in respect to the first 24 h of manufacture. They suggest that cheese is the most interesting and challenging dairy product due to an inherent instability that results from a series of biochemical changes. Whey is the valuable byproduct derived from the cheesemaking process. Lactose and whey proteins are the main compounds of whey and the main reasons for its valorization, through the production of whey cheeses, functional/nutritional whey proteins concentrate, bioactive peptides, and oligosaccharides [2,3].

In the present article collection, two publications for the Parmigiano Reggiano cheese are included. Considering the demanding cheese making conditions and the long-term ripening of this cheese variety, relevant studies are of particular importance. Franceschi et al. [4] designed a two-year study in various cheese factories to assess the performance of milk with high somatic cell count (SCC), i.e., from 400,000 to 1,000,000, when it is used for the manufacture of Parmigiano Reggiano cheese. Compared to control milk with SCC <400,000 cells/mL, the high SCC milk contained significantly lower casein, phosphorous, and chloride and exhibited lower casein number and titratable acidity. Statistically significant reduction of yield by approximately 8.8% was observed after 24 months of ripening of cheese made from milk with high SCC although cheese moisture was not affected. In fact, the reduction was significant for both actual and dry yield, being in accordance with the significantly higher fat loss—by approximately 20%—which occurred during cheesemaking of high SCC milk. According to the findings, the inferior composition of high SCC milk and the curd behavior made there from, jeopardize among other things, the financial result of this laborious production process. 

D’Incecco et al. [5] studied the effect of extending the ripening period from 12 to 50 months on the Parmigiano Reggiano cheese characteristics. It was shown that in this extra-hard cheese, moisture content decreased constantly throughout the ripening period. Primary proteolysis was due to chymosin and mostly to plasmin activity. Secondary proteolysis revealed high numbers of FAA with glutamic acid being the predominant. Regarding volatile compounds, short chain odd-numbered FFA, esters, and ketones were the most abundant without, however, causing any off-flavor. Cheese microstructure showed that free fat was trapped in the protein network. Moreover, the cheese porosity increased significantly, and tyrosine crystals appeared after the 12 months of ripening. The researchers concluded that both biochemical and structural changes continue to take place in this cheese up to 50 months of ripening and, they suggest that if this fact is communicated to consumers, the ‘long-ripened’ cheese could achieve a better price. 

A particular hard-type Italian PDO cheese named Nostrano Valtrompia manufactured from raw milk partially skimmed by spontaneous creaming and supplemented with saffron is the objective of the article written by Bettera et al. [6]. The article presents the key physicochemical, macrostructural, and sensory properties of this cheese type and compares the effect of a 12 to 16 month ripening period in a non conditioned traditional cellar with ripening in a temperature conditioned warehouse. Interestingly, separate samples were taken from the rind, the underrind and the inner part of cheeses for the estimation of moisture, aw, and color parameters. The moisture gradient from the rind to the center of the cheese, - increasing from 15.3 to 31%, on average—influenced texture, moisture, aw, and color. Although cheeses ripened under the two different conditions shared a common identity, cheeses ripened in a conditioned warehouse exhibited a slightly softer texture, a slightly different porosity distribution, and a different sensory perception compared to traditionally ripened counterparts. Based on their findings, the authors propose the use of new sites in the region that can be used as alternative ripening warehouses, to overcome the “capacity” difficulties in the PDO area.

The objective of the study of Štefániková et al. [7] was the raw or pasteurized soft/creamy sheep milk traditional Bryndza cheese of Slovakia, which has a limited period of ripening, a moisture ranging from 52 to 56%, and a pH from 5.1 to 5.3. They examined the effect of the manufacturing way—traditional or industrial—on cheese gross composition, microflora, and volatile organic compounds; the latter by means of e-nose and GC-MS. Season and production conditions affected the features of Bryndza cheese. Presumptive lactococci, followed by presumptive lactobacilli and enterococci were the most dominant bacterial groups, whereas Dipodascus geotrichum was always detected. As expected, the lack of heat treatment of milk dramatically increased coliforms and enterococci counts. Key volatile organic compounds were ethyl acetate, isoamyl acetate, 2-butanone, hexanoic acid, D-limonene, and 2,3-butanedione, significantly affected by the production conditions and season. For example, according to PCA analysis of the e-nose dataset, cheeses produced in May are differentiated from those produced in September.

The results of the analysis of water-soluble extracts of Gouda-type cheeses, made with different starters by nano ultra-high-performance chromatography data-independent acquisition high-resolution mass spectrometry (nanoUHPLC-DIA-HRMS), are presented in the article of Arju et al. [8]. They proposed a cheese peptide method with a gradient time shorter than usual, by which they identified 558 cheese peptides throughout 90 days of ripening. They concluded that the steps of sample preparation should be further investigated to enhance transmission of shorter peptides related to bioactivity and flavor.

In contrast to ripened cheeses, fresh spreadable cheese is a special cheese category usually derived from the combination of renneting and acid coagulation of the cheese milk. Several technological parameters may affect the characteristics of such a cheese product. Fat-content, homogenization, and heat treatment of the cheese milk for producing Quark-type cheese were studied by Lepesioti et al. [9]. The researchers reported that homogenization increased the moisture and protein retention in cheese, and this was more pronounced in full-fat cheese. Moreover, cheeses made with homogenized milk were softer and less adhesive, gummy, and chewy from those made with unhomogenized milk. Heat treatment also improved both, the moisture and protein retention in cheese. The authors finally concluded that heat treatment under conditions that denature β-Lg and α-La by 80% and 25%, respectively, is applied to the cheese milk, is consistent with the development of an appropriate texture for this cheese type without adding stabilizers; hence, a ‘clean label’ full- or reduced-fat Quark-type cheese can be produced. 

In the framework of the circular economy, whey can be returned to cheese milk to improve the characteristics of reduced fat cheese. This is due to the ability of whey proteins particles to act as fat replacers. Borges et al. [10] used heat treated (90 °C for 20 min) UF concentrated whey and other dairy byproducts, i.e., buttermilk and second cheese whey (Sorelho), in the manufacture of reduced fat wash curd bovine cheese. They found that the fortification of the cheese milk with concentrated whey at a ratio of 5% increased the protein content as well as the ratio of protein in dry matter and fat in dry matter of cheese, which was ripened up to 90 days. In parallel, it increased the pH, the hardness, and chewiness and decreased the adhesiveness of cheese after 30 days of ripening. The researchers reported that further investigation is needed regarding the form of the added byproducts, i.e., liquid or dry form as well the optimization of mixtures of them. 

Hussein et al. [11] investigated the hydrolysis of WPC by alcalase by means of response surface methodology. The aim of the study was to determine the appropriate conditions for the production of protein hydrolysates with dual biofunctionalities, that is, angiotensin-I converting enzyme (ACE) inhibitory and antioxidant activities. The optimized hydrolysis parameters were: temperature 58.2 °C, E/S ratio 2.5%, pH 7.5, and duration 361.8 min. Under these conditions, the experimental results were similar to the respective predicted values, i.e., 89.2% hydrolysis degree vs. theoretical 89.6%, 98.4% ACE-inhibitory activity vs. 98.8%, 50.1% DPPH radical scavenging activity vs. 50.6%, and 73.1% ferrous ion chelating activity vs. 74.0%. The authors suggested that their findings could be further exploited in research studies for the in vivo efficacy of WPC hydrolysate.

Whey is used in beverages to increase their nutritional and biofunctional value. Whey protein isolate (WPI) has high nutritional value because it contains more than 90% whey protein and for this reason it is used in products aiming to special diets. However, WPI in whey beverages with pH 4–6.0 exhibits instability since the pI of whey proteins is around 5.1. Shang et al. [12] achieved to improve the stability of WPI in an aqueous solution at pH 4.5 by conjunction with polysaccharide from Flammulina velutipes, a species of edible mushroom, via noncovalent interactions. This polysaccharide (FVPs) interacting with WPI alters the secondary structure of whey proteins, and through electrostatic interactions with the protein surface, improves the protein stability. The ratio 1:12.5 between FVPs and WPI was reported as the optimum for noncovalent interactions. The complex WPI-FVPs showed increased antioxidant activity regarding the ABTS-, hydroxyl- and superoxide anion-radical inhibition. Moreover, it remained intact during in vitro digestibility by pepsin, while it was completely hydrolyzed by trypsin. The researchers concluded that the WPI-FVPs complex could be used in WPI-based beverages made by the food industry.

León-López et al. [13] investigated the characteristics of whey-based fermented beverages fortified with hydrolyzed collagen at ratios from 0.3% to 1%. It was shown that addition of collagen increased the pH, the viscosity, the nutritional value, and the in vitro bioavailability of the product. Specifically, addition of 1% collagen increased protein content and the antioxidant activity regarding the ABTS and DPPH radical inhibition. Moreover, no pathogen microorganism was detected. The authors proposed that this product could be addressed to specific consumers groups such as elderly people and athletes.

Concluding the presentation of this Special Issue, we would like to thank the above-mentioned research teams for their contributions in the present article collection, which provide excellent examples for the multidisciplinary character of the research on cheese and whey.
